# Media optimization for CHO fed-batch processes using a DoE approach in automated high-throughput single use ambr15 bioreactors

**DOI:** 10.1186/1753-6561-9-S9-P15

**Published:** 2015-12-14

**Authors:** Christophe Grimm, Wolfgang Kusser, Brian Lee, Greg Bremer, Alexis Bossie

**Affiliations:** 1 Lonza Bioscience, Walkersville, MD, 21793, USA; 2 Sartorius Stedim France S.A.S., 13781 Aubagne, France; 3 Sartorius Stedim North America Inc., Bohemia, NY 11716, USA

## Background

Chinese Hamster Ovary (CHO) derived cells are the most commonly used cell lines for the production of biopharmaceuticals. We developed a plug and play method to optimize the growth medium for a given CHO production cell line. The experiment consists of a Mixture Design of Experiments (DOE) approach using different basal media to select the optimized formulations for a specific CHO cell line. The package for media optimization consists of different base media together with the ambr15 high throughput bioreactor and integrated MODDE DoE software.

## Methods and results

**Figure 1:**
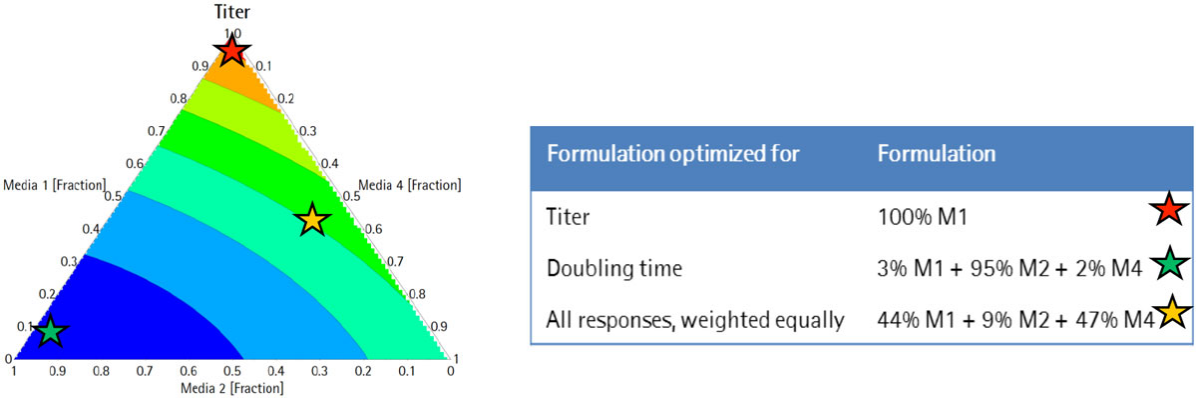
**Contour plot (titer) and optimal mixes: DOE predictions of optimal media mix for titer and media formulations optimized for titer (red star) doubling time (green star) and VCD/Titer/doubling time weighed equally (yellow star) are shown**.

A suspension adapted CHO DG44 cell line expressing a monoclonal antibody was used for the experiments. Media mixes composed of differing concentrations of nutrients were used in a mixture DOE with 20 different media and 3 center points. All conditions were inoculated in the ambr at the same starting density of 2.0xE5 cells/mL. Dissolved oxygen, pH, and temperature control were maintained throughout the batch process. Each bioreactor was sampled for cell count and viability. Metabolites as well as IgG titers were measured. Peak Viable Cell Density (VCD), Doubling Time (DT), and IgG titer results were collected and modeled as responses using MODDE 10 design of experiments (DOE) software to evaluate the optimal media mixtures. Results are shown in Figure 1.

## Conclusions

The ambr15 and MODDE software used here provided a path to obtain effective media formulations for a given specific CHO cell line. The approach consists of a comprehensive service containing four basal media to prepare the mixes and the ambr15. It is supported by an application specialist to guide the experiments and interpretation of results.

